# Millimeter-Wave-to-Terahertz Superconducting Plasmonic Waveguides for Integrated Nanophotonics at Cryogenic Temperatures

**DOI:** 10.3390/ma14154291

**Published:** 2021-07-31

**Authors:** Samane Kalhor, Majid Ghanaatshoar, Hannah J. Joyce, David A. Ritchie, Kazuo Kadowaki, Kaveh Delfanazari

**Affiliations:** 1 Laser and Plasma Research Institute, Shahid Beheshti University, Tehran 19839-69411, Iran; samane.kalhor@gmail.com (S.K.); m-ghanaat@sbu.ac.ir (M.G.); 2 Electrical Engineering Division, University of Cambridge, Cambridge CB3 0FA, UK; hannah.joyce@eng.cam.ac.uk; 3 Department of Physics, Cavendish Laboratory, University of Cambridge, Cambridge CB3 0FA, UK; dar11@cam.ac.uk; 4 Division of Materials Science, Faculty of Pure & Applied Sciences, University of Tsukuba 1-1-1, Tennodai, Tsukuba, Ibaraki 305-8573, Japan; dr.kazuo.kadowaki@gmail.com; 5 James Watt School of Engineering, University of Glasgow, Glasgow G12 8QQ, UK

**Keywords:** Bi_2_Sr_2_CaCu_2_O_8+δ_ quantum material, high-temperature superconductor, on-chip light sources and detectors, plasmonic waveguides, quantum emitters, THz integrated circuitry, cryogenic circuitry

## Abstract

Plasmonics, as a rapidly growing research field, provides new pathways to guide and modulate highly confined light in the microwave-to-optical range of frequencies. We demonstrated a plasmonic slot waveguide, at the nanometer scale, based on the high-transition-temperature (*T_c_*) superconductor Bi_2_Sr_2_CaCu_2_O_8+δ_ (BSCCO), to facilitate the manifestation of chip-scale millimeter wave (mm-wave)-to-terahertz (THz) integrated circuitry operating at cryogenic temperatures. We investigated the effect of geometrical parameters on the modal characteristics of the BSCCO plasmonic slot waveguide between 100 and 800 GHz. In addition, we investigated the thermal sensing of the modal characteristics of the nanoscale superconducting slot waveguide and showed that, at a lower frequency, the fundamental mode of the waveguide had a larger propagation length, a lower effective refractive index, and a strongly localized modal energy. Moreover, we found that our device offered a larger SPP propagation length and higher field confinement than the gold plasmonic waveguides at broad temperature ranges below BSCCO’s *T_c_*. The proposed device can provide a new route toward realizing cryogenic low-loss photonic integrated circuitry at the nanoscale.

## 1. Introduction

The high-transition-temperature (*T_c_*) superconducting Bi_2_Sr_2_CaCu_2_O_8+δ_ (BSCCO) intrinsic Josephson junctions (IJJs)-based THz emitters radiate intense, coherent, and continuous THz photons with frequencies ranging from 0.1 to 11 THz [1,2,3,4,5,6,7,8,9,10,11,12,13,14,15,16,17,18,19,20,21,22,23,24,25,26,27,28]. Such THz devices can also be used as surface current-sensitive detectors due to the unique electrodynamics of the BSCCO quantum material. Therefore, BSCCO-based devices are valuable for many applications, including THz imaging, interferometry, and absorption measurement [29]. The design of low-loss mm-wave-to-THz components, e.g., waveguides, capable of being integrated with such superconducting emitters, and detectors, are vital to accomplishing all BSCCO-made chip-integrated mm-wave-to-THz circuitry. Moreover, the exploitation of superconducting quantum materials into the architectures of waveguides enables the implementation of the real-time sensing and controlling of waveguides, due to the sensitivity of the quantum mechanical phases of superconductors to external stimuli such as magnetic fields, temperature, light, and current [30]. Cooper pairs in superconductors have an equivalent response to that of electrons in plasmonic metals at high frequencies [30]. Plasmonics deals with propagating surface plasmon polaritons (SPPs) such as the coupled oscillation of electrons and electromagnetic waves [31]. The innovative physical effect of plasmonic devices such as subwavelength localization of the electromagnetic field provides a new route in novel chip-scale integrated photonic devices [32,33,34]. In superconductors, the coherent oscillation of plasmonic waves is a result of the formation of Cooper pairs and the absence of scattering [35]. The surface plasmon oscillations in superconducting metals have been extensively investigated for single and multiple-film systems, and microstructure arrays [36,37,38,39,40,41]. It was shown that extraordinary transmission through a subwavelength hole array in a superconducting NbN film arises from the enhancement of SPPs below the transition temperature [39]. Moreover, it was demonstrated that the existence of superconducting plasmons in the YBCO subwavelength hole array is due to the dominance of kinetic resistance over inductance resistance in superconductors [40]. Furthermore, it was shown that YBa_2_Cu_3_O_7_ (YBCO) and niobium (Nb) plasmonic superconducting waveguides offer a superior long plasmon propagation distance in comparison to noble metals at THz frequencies [35,41] due to the intrinsic low-loss plasmonic properties of superconductors.

In this paper, we propose a mm-wave-to-THz superconducting plasmonic slot waveguide (PSW) based on BSCCO. We first studied the modal characteristics of the BSCCO PSW, including the effective refractive index, the propagation loss of SPPs, and mode energy confinement. Furthermore, we investigated thermal tuning of the modal characteristics of such waveguides at temperature ranges between *T* = 10 and 100 K at the selected frequencies of *f* = 0.1, 0.3, 0.5, and 0.8 THz. The proposed waveguide can be integrated with BSCCO-based THz sources and detectors. It is also suitable for various applications such as tunable modulators and photodetectors.

## 2. Structure Design and Methods

**Figure 1:**
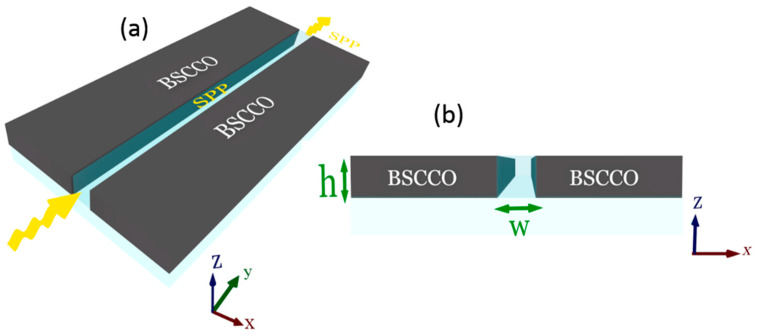
(**a**) Three-dimensional schematic diagram of the proposed suspended BSCCO-based plasmonic slot waveguide, (**b**) and the cross-sectional view of the waveguide at the *x-z* plane.

A 3D schematic of the proposed BSCCO PSW is shown in Figure 1a. The cross-sectional view of the 3D SPW at the *x-z* plane in Figure 1b shows that the waveguide consists of a deep subwavelength air slot of width *w* in a thin film of BSCCO with a thickness of *h*. The generated SPPs propagate through the air slot. 

We employed the numerical finite element simulation method (FEM)-based mode solver to calculate the eigenmodes of the plasmonic waveguide at a specific frequency ω. Here, an exp(−iβy) dependence for the electric field was considered because the waveguide is uniform along the *y*-direction [42]. Therefore, the electric field *E* distribution in the waveguide can be written as

(1) E(x,y,z)=E(x,z)exp(−iβy)

where $\beta =\beta_{1}+i\beta_{2}$ is the complex propagation constant of the waveguide’s mode.

The electromagnetic wave equation is defined as [43]

(2) $$\nabla \times \nabla \times E=\frac{\omega^{2}}{c^{2}}\varepsilon E$$

where c is the speed of light in vacuum. For our waveguide whose structure is uniform in the *y*-direction, the wave equation reduces to [44]

(3) $$\nabla_{⊥}^{2}E+\left(\frac{\omega^{2}}{c^{2}}\varepsilon -\beta^{2}\right)E=0$$

where we used the definition $\nabla_{⊥}^{2}=\partial^{2}/\partial x^{2}+\partial^{2}/\partial z^{2}$.

By calculating the eigenvalue of Equation (3), the propagation constant β is obtained. Then, the modal characteristics of the waveguide, including the real part of the effective refractive index (*N_eff_*) and propagation length (*L_p_*) of SPPs for the fundamental mode of the BSCCO PSW, can be calculated from equations [42,45]:

(4) $$N_{eff}=\beta /k_{0},$$

(5) $$L_{p}=1⁄\left(2\;\mathrm{Imag}\left(\beta \right)\right),$$

where $k_{0}$ is the free-space wavevector. *N_eff_* is an indicator of the localization of SPP’s energy and wavelength. In addition, the dispersion relation of the waveguide is defined as ω=ω(β) [42].

In Equation (2), ε is the permittivity of the relevant medium. The permittivity of air $\varepsilon_{air}$ is 1, and the temperature- and frequency-dependent *a-b* plane complex conductivity of BSCCO film with *T_c_* = 85 K is extracted from the experimental THz time-domain spectroscopy data [46,47,48]. The complex permittivity of BSCCO can be obtained from its complex conductivity [48].

For obtaining the eigenmode of the waveguide, the area of computation is considered large enough, and the perfectly-matched-layer (PML) absorbing boundary conditions are used along the *x*- and *z*-axis. Therefore, the reflection of fields from the boundaries is negligible. In addition, the factor of mode confinement is calculated as a ratio of power flow in the slot (w*h area) to the total power flow in the waveguide normal to the *x-z* plane [45].

(6) $$\Gamma =\frac{\int_{slot}Re\left\{\left(E\times H^{*}\right)\cdot n\right\}\;dA}{\int_{total}Re\left\{\left(E\times H^{*}\right)\cdot n\right\}\;dA}$$

where power flow is $S_{n}=\left(1/2\right)Re\left\{\left(E\times H^{*}\right)\cdot n\right\}$. Here, *E* and $H^{*}$ are the electric and complex conjugates of magnetic field vectors, respectively, and *n* is the normal unit vector in the *y*-direction.

## 3. Results and Discussion

**Figure 2:**
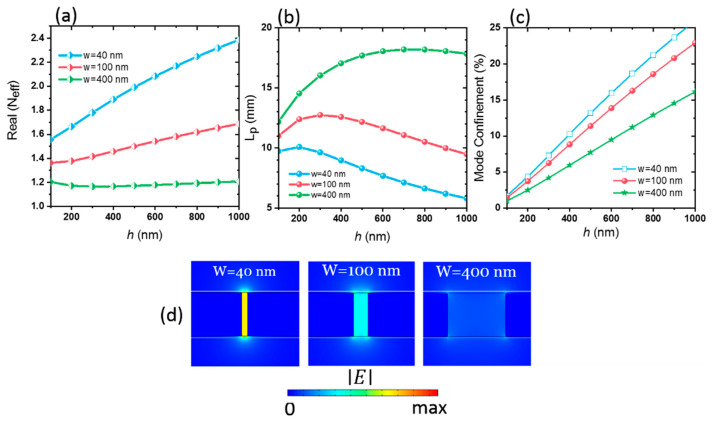
(**a**) Real part of the mode effective refractive index (*N_eff_*), (**b**) propagation length (*L_p_*), and (**c**) mode confinement of BSCCO THz plasmonic waveguide as a function of BSCCO thickness *h* for three slot widths of *w* = 40, 100, and 400 nm at *f* = 0.1 THz and *T* = 10 K. (**d**) Electric field distribution at slot widths of *w* = 40, 100, and 400 nm for BSCCO height *h* = 300 nm. All field distributions curves have the same color bar.

The highest possible mode quality of the waveguide was obtained through optimization of the slot width *w* and BSCCO thin-film thickness *h* at the temperature *T* = 10 K and frequency *f* = 0.1 THz. The modal characteristics are controllable by the structural size of the waveguide. The effective refractive index (*N_eff_*) and propagation length (*L_p_*) of the BSCCO PSW as a function of BSCCO height h for different slot widths w are shown in Figure 2a,b.

At each BSCCO/air interface within the air gap, SPPs are formed. These two formed SPPs are coupled and create a transverse electromagnetic (TEM) wave mode. *N_eff_* is larger than the air refractive index and is adjustable by the structural size. As h increases, *N_eff_* increases, but *L_p_* decreases because the superconductor/air interface height in the slot increases. The larger portion of BSCCO (whose *N_eff_* is higher than air) results in a larger *N_eff_.* Besides, as width w decreases, *N_eff_* increases, and *L_p_* reduces. Once the slot width is narrow, the SPP related to the two BSCCO surfaces form the coupled SPPs [49]. Therefore, as the w of the slot decreases, the propagation constant β increases and leads to the increase in *N_eff_* and reduction in *L_p_* [42]. The fall of *L_p_* at very low *h* arises from the decoupling of two formed SPPs.

The mode confinement of SPPs is shown in Figure 2c. It determines the enhancement of energy in the slot region. The mode confinement reduces with increasing slot width w. To clarify this, we show the electric field distribution at different slot widths *w* for a constant *h* = 300 nm in Figure 2d. We see that the slot width of *w* = 400 nm has the lowest electric field distribution within the slot. Even though the narrower slot dimension has a large energy confinement, it suffers from the lower propagation length. 

There is a tradeoff between the energy confinement of SPPs within the slot and SPP’s propagation length. The largest *L_p_* for SPW with thickness h = 300 nm is for a slot width of w = 100 nm. Hence, these values (w = 100 nm and h = 300 nm) were chosen as SPW optimum dimensions. Based on this optimization, *N_eff_* is 1.42. The mode has a shorter wavelength in comparison to the free space. Therefore, the SPP’s wavelength, which is defined as $\lambda_{0}/N_{eff}$, is 2.1 mm, and the SPP’s field is confined in the air slot as small as $\lambda_{0}^{2}/(3\times 10^{8})$. Here, $\lambda_{0}$ = 3 mm is the free-space wavelength. The propagation distance of SPPs is 12.73 mm, which is equal to six effective wavelengths.

**Figure 3:**
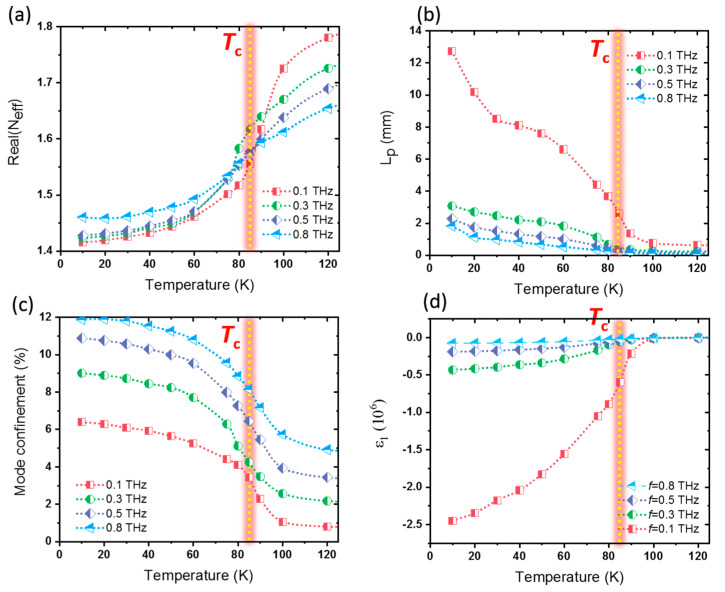
(**a**) Real part of the mode effective refractive index (*N_eff_*), (**b**) propagation length of SPPs (*L_p_*), (**c**) the mode confinement of the PSW as a function of temperature, and (**d**) the real part of BSCCO permittivity at different frequencies. The vertical dashed lines show the *T_c_* of BSCCO.

The modal characteristics of the waveguide are dependent on the plasmonic properties of BSCCO. *N_eff_*, *L_p_*, and mode confinement are shown in Figure 3a–c as a function of temperature for four different frequencies of *f* = 0.1, 0.3, 0.5, and 0.8 THz. For each frequency, it is found that *N*_eff_ reduces but *L_p_* and mode confinement increase significantly as BSCCO enters the superconducting state below *T_c_* (the vertical dashed line). The magnitude of the real part of BSCCO permittivity ($\left|\varepsilon_{1}\right|$) increases below *T_c_* (see Figure 3d). The continuity of the normal component of the electric field displacement (D) at the boundary of the BSCCO and air interface as $\varepsilon_{BSCCO}E_{BSCCO\;⊥}=\varepsilon_{air}E_{air\;⊥}$ results in the decrease in the electric field in BSCCO, by increasing $\left|\varepsilon_{1}\right|$, due to the material temperature reduction. Here, $\varepsilon_{BSCCO}$ and $\varepsilon_{air}$ are the permittivities of BSCCO and air, respectively. $E_{1⊥}$ and $E_{2⊥}$ are normal components of the electric field in BSCCO and air, respectively. The electric field reduction in the BSCCO results in a lower modal propagation constant *β* and lower *N_eff_*. The growth in *L_p_* and mode confinement with the cooling of the waveguide is also the outcome of lower *β*.

Figure 3d shows the real part of the BSCCO permittivity at different frequencies. Indeed, superconductors are intrinsically plasmonic media with a negative real part of complex permittivity. Superconductor plasmonic properties are determined by the coexistence of normal and superconducting plasma. Normal carriers are responsible for scattering processes. At *T_c_* and above, all carriers are in the normal phase. With a reduction in temperature to below *T_c_*, the ratio of superconducting carriers to normal carriers increases. At zero temperature, all carriers turn into supercarriers according to the well-known two-fluid model [40,50]. Above *T_c_* (vertical dashed line in Figure 3d) in the normal state of BSCCO, $\varepsilon_{1}$ has a very low value. The material is nevertheless in the plasmonic regime, with a significant loss. At cryogenic temperatures below *T_c_* of BSCCO, the absolute value of $\varepsilon_{1}$ increases. Therefore, loss of the material decreases due to the growth of supercarrier densities. The growth of SPP’s propagation length by reducing the temperature in Figure 3b is a result of loss reduction in BSCCO below *T_c_*.

From Figure 3a–c, it could also be found that *N_eff_* increases and *L_p_* decreases with frequency. The growth in *N_eff_* (Figure 3a) is due to the reduction in the absolute value of the real part of BSCCO permittivity with frequency (see Figure 3d), and also the larger penetration of mode power in BSCCO. Larger modal penetration in BSCCO means lower mode confinement within the slot (see Figure 3c). The reduction in *L_p_* with frequency in Figure 3b is a result of the increasing Ohmic loss and also due to the larger fraction of the modal power in BSCCO. However, there are less prominent differences between waveguide characteristics below and above the transition temperature at higher frequencies. This is first due to the increase in loss rate at higher frequencies. Moreover, in BSCCO, the two-fluid model cannot explain the low-frequency conductivity, as well as that of other layered superconductors such as YBCO with less anisotropy. In BSCCO, as an anisotropic cuprate, there is an additional spectral weight at low frequencies that increases as the material is cooled toward zero. This residual spectral weight (the so-called collective mode) arises from the fluctuation in the condensate order parameters [46,47,48] and draws about 30% of the spectral weight from the condensate [46]. However, there is good agreement between the BSCCO conductivity and the two-fluid model at higher frequencies.

**Figure 4:**
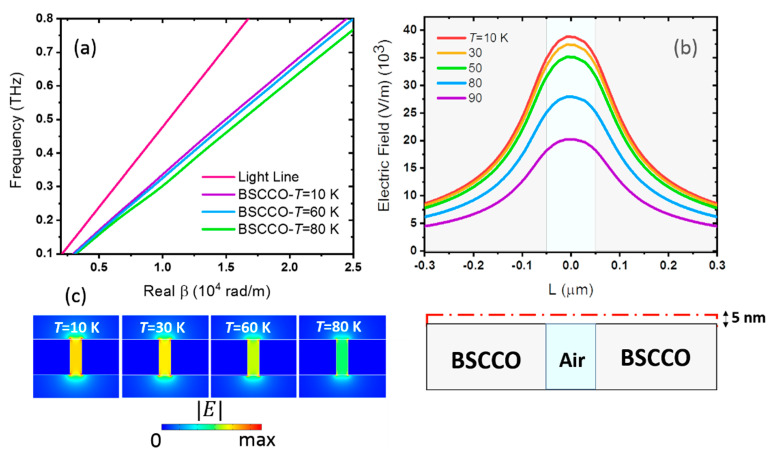
(**a**) Dispersion curves for the mode of the BSCCO PSW at *T* = 10 K (purple), *T* = 60 K (blue), and *T* = 80 K (green). The red line shows the dispersion relation of the THz waves propagating in free space. (**b**) Electric field intensity at the red dashed line 5 nm above the waveguide, at different temperatures between *T* = 10 and 90 K, at *f* = 0.1 THz. The cross-section of the waveguide shows the relative position of the BSCCO film and the slot area in the electric field curves. The pale blue area shows the gap, while the grey area shows the BSCCO part. (**c**) The electric field distribution of the BSCCO THz PSW for width *w* = 100 nm and height *h* = 300 nm at *f* = 0.1 THz for selected temperatures *T* = 10, 60, and 80 K. All field distributions have the same color bar.

For clarifying the effect of temperature, the dispersion relation of the mode of BSCCO PSW is shown in Figure 4a. Compared to the light line (dispersion relation of vacuum, shown with red color), it infers that the waveguide supports a bound mode because the waveguide lines are on the right side of the light line [51]. With the reduction in temperature, the slope of the curves becomes sharper. Therefore, cooling the waveguide results in reducing the refractive index. Lower *N_eff_* for lower temperature dictates shorter SPP wavelengths. Moreover, the dispersion curves show that the energy is less confined at lower frequencies due to approaching the light line.

For further investigation of the thermal tuning of confinement of the modal energy within the slot, the electric field distribution at 5 nm above the waveguide’s surface along the dashed line is shown in the bottom panel of Figure 4b, for selected temperatures, at f = 0.1 THz, and for the optimized waveguide size. Here, the top curve is aligned vertically with respect to the cross-sectional view of the waveguide. The pale blue area in the schematic of the waveguide and within the curve of Figure 4b shows the air slot area, while the grey area shows the BSCCO thin-film area. The electric field is enhanced within the slot for all temperatures, whereas it grows as temperature reduces due to the reduction in the loss rate. The electric field distribution at different temperatures in Figure 4c also shows the enhancement of the field within the slot with the reduction in temperature. 

**Figure A1:**
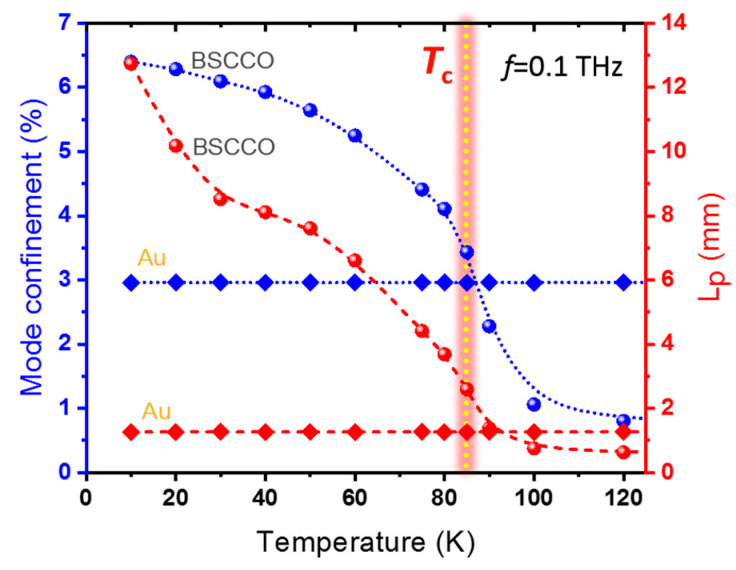
Mode confinement and propagation length of SPPs (*L_p_*) of BSCCO and gold plasmonic waveguide with structural size of h = 300 nm and w = 300 nm at f = 0.1 THz. The vertical dashed line shows the *T_c_* of BSCCO. Modal properties of gold waveguide are not dependent on temperature.

The comparison between the BSCCO waveguide and gold waveguide with the same structural size of *h* = 300 nm and *w* = 100 nm at the frequency of *f* = 0.1 THz shows that the BSCCO waveguide has a larger energy confinement and larger propagation length below *T_c_* (see Figure A1). Therefore, the propagation characteristics of the proposed BSCCO waveguide are better than those of the gold waveguide below *T_c_*. Above *T_c_*, the propagation length of SPPs for the BSCCO waveguide is comparable to that of the gold plasmonic waveguide (see Figure A1 in Appendix).

**Figure A2:**
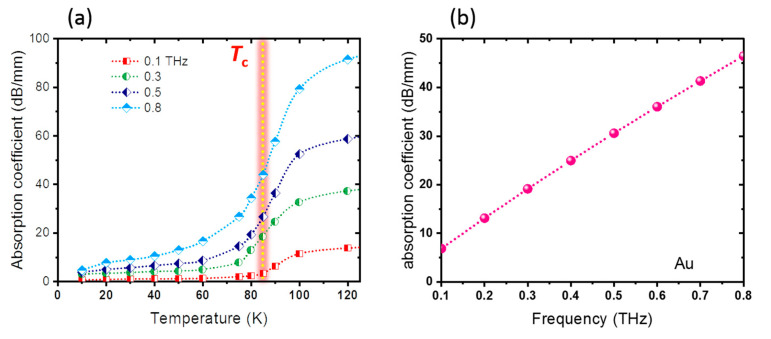
Absorption coefficient of (**a**) BSCCO plasmonic waveguide as a function of temperature at different frequencies. The vertical dashed line shows the *T_c_* of BSCCO. (**b**) Gold plasmonic waveguide as a function of frequency. The absorption coefficient of the gold waveguide is temperature-independent.

The absorption coefficient (α) of the waveguide can be calculated from α=2 Imag(β) [52]. The absorption coefficients of BSCCO and the gold plasmonic waveguide as a function of temperature and frequency are shown in Figure A2. For the BSCCO waveguide at the frequency of *f* = 0.1 THz, the absorption coefficient is equal to 0.68 dB/mm at *T* = 10 K and it increases to 3.34 dB/mm at *T* = 85 K (the *T_c_* of BSCCO). For gold, the absorption coefficient is as high as 6.85 dB/mm at the frequency of *f* = 0.1 THz. Nevertheless, the absorption coefficient of the BSCCO waveguide is comparable to the gold waveguide above *T_c_*. Additionally, the absorption confinement of both waveguides increases with frequency as a result of increasing Ohmic losses.

## 4. Conclusions

We numerically investigated the temperature-dependent modal characteristics of a high-T_c_ superconducting BSCCO plasmonic slot waveguide, including the refractive index, propagation length, and the mode confinement in the slot region at the mm-wave-to-THz range of frequencies. We showed that the propagation length of SPP increases as material enters the superconducting phase. In addition, we investigated the frequency tuning of the modal characteristics. Compared with the gold waveguide, the BSCCO waveguide at T = 10 K offers higher mode confinement within the gap and a larger propagation length below T_c_. The proposed BSCCO plasmonic waveguide helps realize a fully integrated BSCCO THz circuitry for applications in cryogenic on-chip quantum communication and low-loss data processing.

## Data Availability

Data are available at this placeholder DOI: https://doi.org/10.17863/CAM.73576.

## Appendix A

The conductivity of gold ($\sigma_{Au}$) is described by the Drude model expression as

(A1) $$\sigma_{Au}=\varepsilon_{0}\frac{\omega_{p}^{2}}{\gamma +i\omega }$$

where plasma frequency $\omega_{p}$ is 2π×2175 THz and collision frequency γ is 2π×6.5 THz [1]. Here, $\varepsilon_{0}$ is the vacuum electric constant. It should be noted that no temperature tuning is expected for the gold waveguide.
